# Responder discordance between migraine frequency and patient-reported outcomes after CGRP-pathway monoclonal antibody treatment: a secondary analysis of public patient-level data

**DOI:** 10.1186/s12883-026-05074-2

**Published:** 2026-06-15

**Authors:** Chuntao Wu, Junwei Wang, Shuai Zhao, Yinxin Wang, Tong Wu

**Affiliations:** 1 Department of Neurology, The First Affiliated Hospital of Henan Medical University, No.88 Jiankang Road, Weihui, Henan 453100 China; 2 Department of Neurosurgery, The First Affiliated Hospital of Henan Medical University, Weihui, Henan China; 3 Department of Otolaryngology, The First Affiliated Hospital of Henan Medical University, Weihui, Henan China; 4 Department of Otorhinolaryngology, Head and Neck Surgery, The First Affiliated Hospital of Nanchang University, Nanchang, Jiangxi China; 5 Department of Otolaryngology, The First Affiliated Hospital of Xinjiang Medical University, Urumqi, Xinjiang China

**Keywords:** Migraine, CGRP monoclonal antibody, Responder definition, Monthly migraine days, MIDAS, HIT-6, Patient-reported outcome, Secondary analysis

## Abstract

**Background:**

Monoclonal antibodies targeting the calcitonin gene-related peptide (CGRP) pathway are established preventive treatments for migraine. Although treatment response is often summarized by reduction in monthly migraine days (MMD), patients and clinicians also judge benefit by changes in disability and headache impact. We examined how often frequency-based and patient-reported outcome measure (PROM) responder definitions classify the same individuals after CGRP-pathway monoclonal antibody treatment and characterized the direction of discordant response patterns.

**Methods:**

We performed a secondary analysis of a public patient-level dataset including 417 patients treated with a first CGRP-pathway monoclonal antibody. Response was defined using source-study criteria: subtype-specific MMD response, Migraine Disability Assessment (MIDAS) response, and Headache Impact Test-6 (HIT-6) response. We quantified response rates, pairwise agreement, directional discordance, and continuous-change correlations. Exploratory logistic regression examined baseline features associated with discordant classification.

**Results:**

MMD, MIDAS, and HIT-6 response occurred in 227/417 (54.4%), 243/417 (58.3%), and 254/417 (60.9%) patients, respectively. Only 131/417 (31.4%) met all three response definitions, and 207/417 (49.6%) had discordant classification across the three endpoints. Observed agreement between MMD and MIDAS response was 66.4% with kappa 0.32, and observed agreement between MMD and HIT-6 response was 68.6% with kappa 0.36. When MMD was compared with any PROM response, PROM-only response occurred in 111/417 (26.6%) patients and frequency-only response in 18/417 (4.3%). Correlations between continuous improvements in frequency-based and patient-reported outcomes were moderate rather than strong.

**Conclusions:**

After CGRP-pathway monoclonal antibody treatment, responder classification depended on whether response was defined by migraine frequency, disability, or headache impact. PROM-only response was more frequent than frequency-only response, suggesting that some patients may report meaningful improvement in disability or impact even when they do not cross diary-based frequency thresholds. These findings do not establish a new responder definition or show that one endpoint is superior; they support complementary reporting of headache frequency and patient-reported burden.

**Trial registration:**

Not applicable.

**Supplementary Information:**

The online version contains supplementary material available at 10.1186/s12883-026-05074-2.

## Background

Migraine is a common and disabling neurological disorder with substantial individual and health-system burden [1]. Calcitonin gene-related peptide (CGRP)-pathway monoclonal antibodies are established options for migraine prevention. The American Headache Society now considers CGRP-targeting therapies a first-line preventive option, and European guidance also supports their use in migraine prevention [2, 3]. Their efficacy has been shown in placebo-controlled trials of erenumab and galcanezumab in episodic and chronic migraine [4–7]. Their growing use has made treatment evaluation a practical issue: patients, clinicians, payers, and trialists all need a way to decide whether treatment has helped enough to continue.

The most familiar summary is reduction in monthly migraine days (MMD). This endpoint is diary-based, clinically intuitive, and embedded in migraine trial guidance and responder-rate conventions [8–10]. However, headache frequency is only one domain of migraine burden. The Migraine Disability Assessment (MIDAS) and Headache Impact Test-6 (HIT-6) are widely used patient-reported outcome measures (PROMs) of disability and headache impact, and their measurement properties and clinically meaningful change thresholds have been studied in migraine populations [11–16]. The Kiel source study used MMD, MIDAS, and HIT-6 to evaluate treatment success after CGRP-pathway monoclonal antibody therapy [17].

MIDAS and HIT-6 are related, but they are not duplicate measures. MIDAS primarily quantifies days of missed or reduced activity attributable to headache, whereas HIT-6 captures perceived headache impact across functional, social, cognitive, and emotional domains. These instruments may therefore improve in parallel with migraine frequency in some patients but diverge in others. This distinction matters when a single binary responder label is used to summarize treatment benefit after CGRP-pathway monoclonal antibody treatment.

The Kiel public dataset provides an opportunity to examine this issue at the patient level because the same treated patients were assessed across frequency, disability, and impact domains [17, 18]. The source study compared response rates and made the individual-level data public. That creates a more specific clinical question: when a patient is labelled a responder after CGRP-pathway monoclonal antibody treatment, does that label identify the same individual across frequency-based and patient-reported endpoints?

The Göbel source study compared responder rates across MMD, MIDAS, and HIT-6 and concluded that PROMs can capture therapeutic benefit not reflected in MMD reduction [17]. Our re-analysis adds a patient-level measurement layer that was not the focus of the source paper: observed agreement, kappa, positive agreement, Jaccard overlap, directional imbalance between PROM-only and frequency-only response, and continuous cross-domain correlations. This reframes the question from whether one endpoint yields a higher group responder rate to whether a binary responder label identifies the same individual across frequency, disability, and headache-impact domains. All analyses addressing this main question used the Kiel patient-level dataset; the fMRI-related Dryad cohort was retained only as a supplementary diary-only example because it lacks MIDAS and HIT-6 and cannot test cross-domain agreement [19–21].

## Methods

### Study design and data sources

This was a secondary analysis of public, anonymized patient-level data, reported with reference to Strengthening the Reporting of Observational Studies in Epidemiology (STROBE) principles where applicable [22]. The main dataset was the Kiel CGRP antibody prophylaxis dataset, which includes 417 patients with episodic or chronic migraine who received a first CGRP-pathway monoclonal antibody at the Kiel Migraine and Headache Center between January 2020 and June 2023 [17, 18]. The dataset includes diagnosis subtype, age, sex, baseline and follow-up monthly migraine days (MMD), monthly headache days (MHD), Migraine Disability Assessment (MIDAS), and Headache Impact Test-6 (HIT-6). Migraine subtype followed the source dataset and was consistent with International Classification of Headache Disorders, third edition (ICHD-3) conventions [23]. The source study required complete documentation for MMD, MIDAS, and HIT-6.

The original Kiel study was approved by the Ethics Committee of Kiel University and patients provided consent for scientific use of routinely collected clinical data [17, 18]. No new patient contact or identifiable data analysis was performed in the present secondary analysis.

The Kiel dataset was used as the primary dataset because it contained patient-level MMD, MIDAS, and HIT-6 measures in the same patients after initiation of a first CGRP-pathway monoclonal antibody. The fMRI-related Dryad dataset was not used as a validation cohort and is described only in the Supplementary material. Its public Dryad record includes the clinical file clinicalData_erenumab_galcanezumab_publication_final_HB.xlsx and a README [20]. The 53 diary records comprise 26 galcanezumab-treated patients from the Basedau fMRI study and 27 erenumab-treated patients from the earlier Ziegeler fMRI study; the erenumab diary variables used for the supplementary analysis were therefore publicly available in the Dryad clinical file associated with reference [20], although the original erenumab imaging study was published separately [21]. Because this dataset differed from the Kiel cohort in design, sample size, follow-up interval, and available outcomes, and because it did not include MIDAS or HIT-6, it was used only to illustrate early headache-day response heterogeneity in the Supplementary material, not to compare agents or extend the Kiel PROM findings.

### Response definitions

Response definitions followed the Kiel source study [17]. MMD response was defined as a reduction of at least 50% in episodic migraine and at least 30% in chronic migraine, consistent with the way subtype-specific frequency response is commonly handled in migraine prevention research [8–10]. Monthly headache day (MHD) response used the same subtype-specific thresholds. MIDAS response was defined as at least 30% reduction among patients with baseline MIDAS greater than 20, a threshold supported by recent registry-based MIDAS work [16]. HIT-6 response was defined as an improvement of at least 5 points, consistent with prior work on clinically relevant HIT-6 change in migraine [14, 15].

These thresholds were retained for comparability with the source study and with prior responder-rate conventions. They should not be interpreted as biologically equivalent across MMD, MIDAS, and HIT-6. Each threshold is an operational cut point imposed on a continuous outcome, and dichotomization can create apparent disagreement among patients whose continuous changes are clinically similar but fall on different sides of a cutoff.

For the primary directional analysis, PROM response was defined as response on MIDAS or HIT-6. Four mutually exclusive classes were then created: both-domain response (MMD response and at least one PROM response), frequency-only response (MMD response without MIDAS or HIT-6 response), PROM-only response (MIDAS or HIT-6 response without MMD response), and neither-domain response. This classification was used as a descriptive map, not as a proposed decision rule.

### Statistical analysis

Categorical results are reported as counts and percentages with Wilson 95% confidence intervals [24]. Pairwise agreement between responder definitions was described using observed agreement, discordance, Cohen kappa with bootstrap 95% confidence intervals, positive agreement, and the Jaccard index [25]. The direction of discordance was assessed by comparing frequency-only and PROM-only response using an exact paired test.

Continuous outcome changes were calculated so that larger positive values represented greater improvement. Associations among continuous improvements in MMD, MHD, MIDAS, and HIT-6 were examined using Spearman correlations to complement the binary responder analyses. Exploratory logistic regression models assessed baseline features associated with discordant classification and with PROM-only response. Candidate predictors were age, sex, baseline MMD, baseline MIDAS, baseline HIT-6, and chronic versus episodic migraine. These models were used to describe associations and were not developed or validated for clinical prediction.

Threshold sensitivity analyses repeated the directional classification using alternative frequency and PROM definitions. These analyses were used to examine whether the main pattern depended entirely on one selected cutoff rather than to select a preferred threshold.

## Results

### Main public cohort

The main analysis included 417 patients, of whom 197 had episodic migraine and 220 had chronic migraine. Mean age was 46.2 years, and 374/417 patients (89.7%) were female. Baseline burden was high, with mean MMD 14.3, mean MHD 16.9, mean MIDAS 100.5, and mean HIT-6 66.9 (Table 1). This profile is broadly consistent with high-burden specialty-care migraine populations receiving CGRP-pathway monoclonal antibodies, although the single-centre setting and marked female predominance should be considered when judging generalizability.

**Table 1:** Baseline characteristics of the main public cohort

Group	*N*	Age, years	Female sex	Baseline MMD	Baseline MHD	Baseline MIDAS	Baseline HIT-6
Overall	417	46.2 (12.8)	374/417 (89.7%)	14.3 (6.0)	16.9 (7.2)	100.5 (70.4)	66.9 (5.1)
Episodic migraine	197	46.4 (11.7)	181/197 (91.9%)	10.3 (2.2)	10.9 (2.6)	72.4 (53.8)	66.1 (5.5)
Chronic migraine	220	46.0 (13.7)	193/220 (87.7%)	18.0 (5.9)	22.3 (5.7)	125.7 (73.9)	67.5 (4.5)

Values are mean (SD) unless otherwise indicated

*MMD* Monthly migraine days, *MHD* Monthly headache days, *MIDAS* Migraine Disability Assessment, *HIT-6* Headache Impact Test-6

### Responder rates and patient-level overlap

**Fig. 1:**
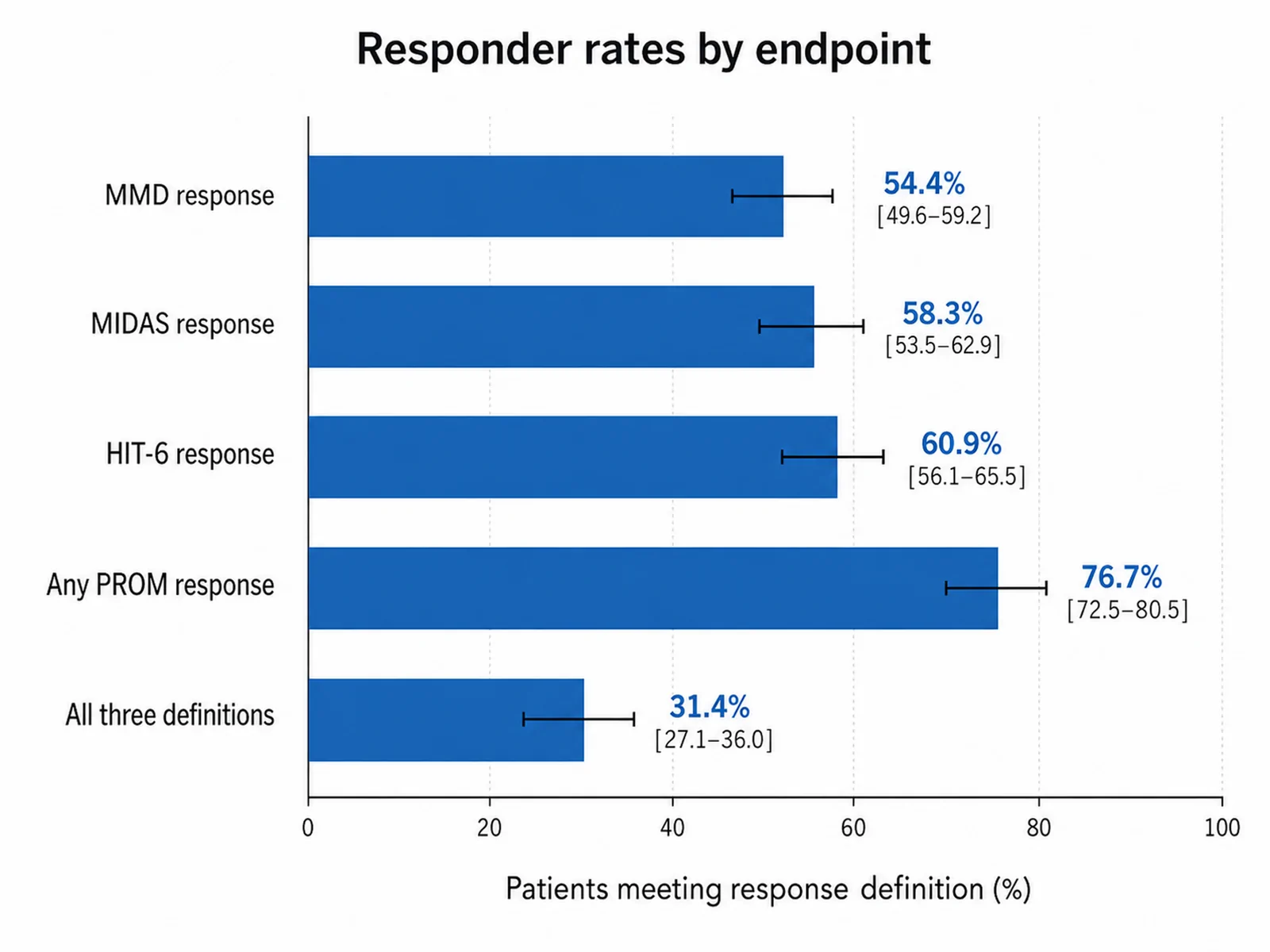
Responder rates by endpoint definition. Bars show the percentage of patients meeting each response definition; error bars show Wilson 95% confidence intervals. PROM response was defined as MIDAS response or HIT-6 response. MMD, monthly migraine days; MIDAS, Migraine Disability Assessment; HIT-6, Headache Impact Test-6; PROM, patient-reported outcome measure

Responder rates were similar in magnitude, but the same patients were not consistently identified across endpoints. MMD response occurred in 227/417 patients (54.4%), MIDAS response in 243/417 (58.3%), and HIT-6 response in 254/417 (60.9%) (Table 2; Fig. 1). At the patient level, 131/417 patients (31.4%) met all three response definitions. Nearly half of the cohort, 207/417 patients (49.6%), had discordant classification across MMD, MIDAS, and HIT-6 (Supplementary Table S1).

**Table 2:** Responder rates by endpoint definition

Definition	Responders
MMD response (source-study subtype-specific threshold)	227/417 (54.4%; 95% CI 49.6–59.2)
MHD response (source-study subtype-specific threshold)	199/417 (47.7%; 95% CI 43.0-52.5)
MIDAS response	243/417 (58.3%; 95% CI 53.5–62.9)
HIT-6 response	254/417 (60.9%; 95% CI 56.1–65.5)
Any PROM response (MIDAS or HIT-6)	320/417 (76.7%; 95% CI 72.5–80.5)
Both PROM responses	177/417 (42.4%; 95% CI 37.8–47.2)
Response by MMD, MIDAS, and HIT-6	131/417 (31.4%; 95% CI 27.1–36.0)

Wilson 95% confidence intervals are shown. PROM response was defined as MIDAS response or HIT-6 response

### Agreement and direction of discordance

**Fig. 2:**
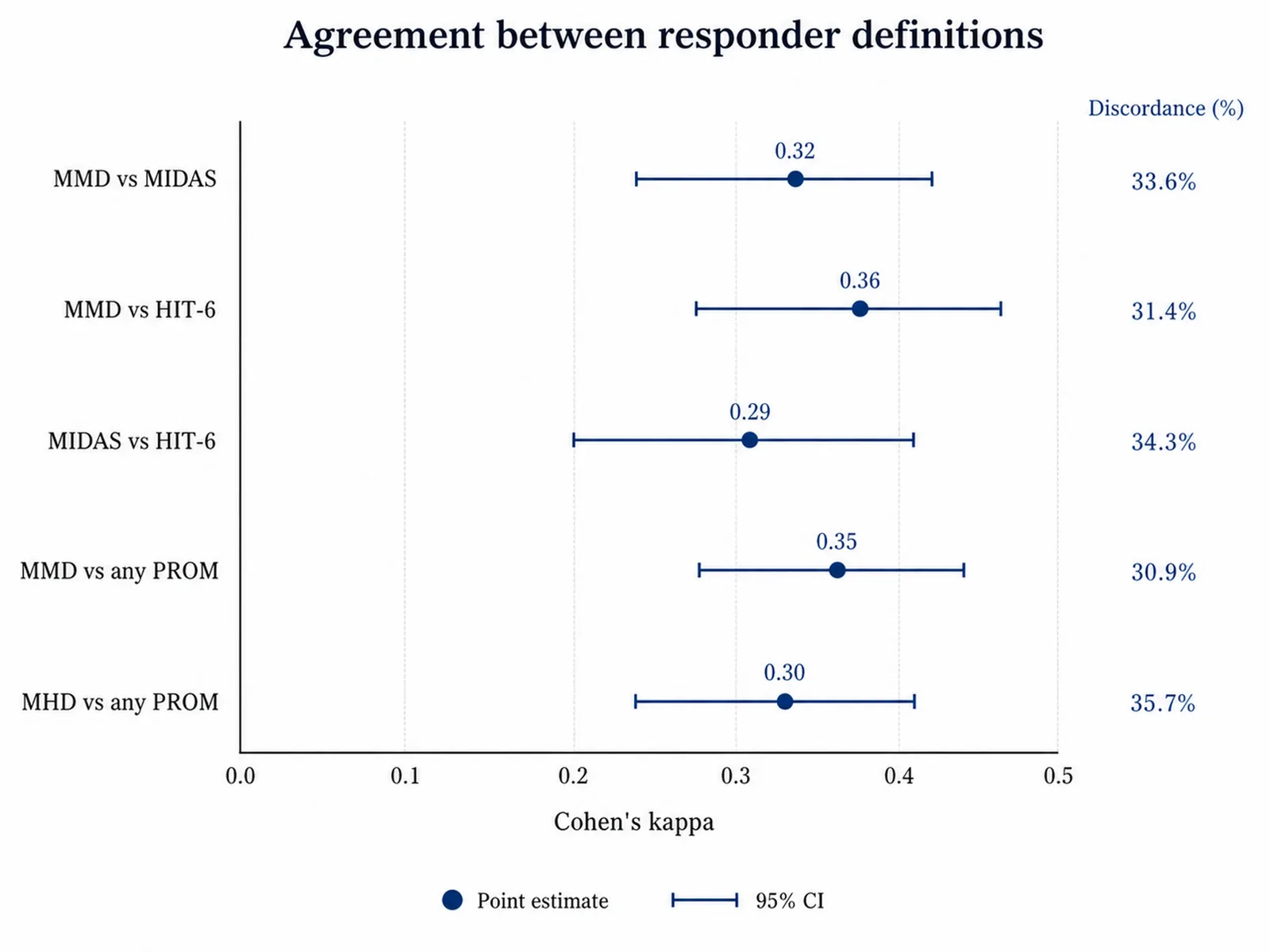
Agreement between responder definitions. Points show Cohen kappa; text gives the discordance rate for each endpoint pair

Agreement between frequency-based and patient-reported response was incomplete rather than absent. MMD response agreed with MIDAS response in 277/417 patients and with HIT-6 response in 286/417 patients. Kappa values were 0.32 for MMD versus MIDAS and 0.36 for MMD versus HIT-6. Agreement between MMD response and any PROM response was also incomplete (kappa 0.35; Table 3; Fig. 2). Although the kappa values were in the low range, observed agreement (66.4–69.1%) and positive agreement (0.70–0.76) indicate substantial overlap; kappa is prevalence-sensitive and was depressed here by asymmetric responder prevalences, especially the high prevalence of PROM response, so it should be interpreted alongside observed and positive agreement. 

**Table 3:** Agreement between responder definitions

Endpoint pair	Observed agreement	Discordance	Cohen kappa (bootstrap 95% CI)	Positive agreement	Jaccard index
MMD vs. MIDAS	277/417 (66.4%)	140/417 (33.6%)	0.32 (0.23–0.40)	0.70	0.54
MMD vs. HIT-6	286/417 (68.6%)	131/417 (31.4%)	0.36 (0.27–0.45)	0.73	0.57
MIDAS vs. HIT-6	274/417 (65.7%)	143/417 (34.3%)	0.29 (0.20–0.38)	0.71	0.55
MMD vs. any PROM	288/417 (69.1%)	129/417 (30.9%)	0.35 (0.27–0.43)	0.76	0.62
MHD vs. any PROM	268/417 (64.3%)	149/417 (35.7%)	0.30 (0.23–0.38)	0.71	0.55

Kappa confidence intervals were obtained with bootstrap resampling

*PROM* Patient-reported outcome measure

**Fig. 3:**
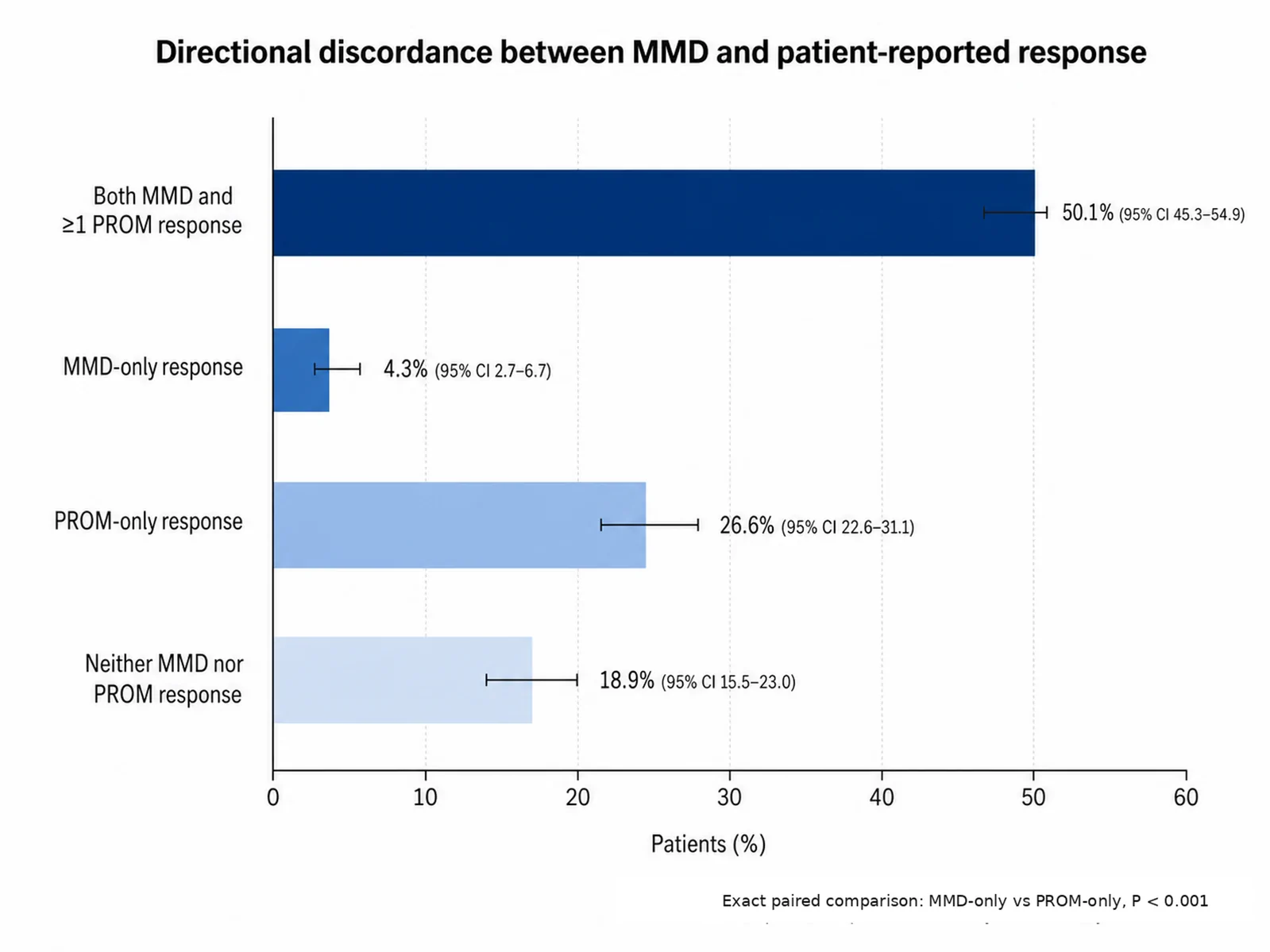
Directional classification between MMD response and patient-reported response. Error bars show Wilson 95% confidence intervals. PROM response was defined as MIDAS response or HIT-6 response

The direction of disagreement was not balanced. When MMD response was compared with any PROM response, 209/417 patients (50.1%) were responders in both domains, 79/417 (18.9%) were responders in neither domain, 18/417 (4.3%) had frequency-only response, and 111/417 (26.6%) had PROM-only response (Table 4; Fig. 3). The difference between PROM-only and frequency-only classification was large in the paired comparison (*P* < 0.001). This directional imbalance motivated the expanded clinical interpretation of PROM-only and frequency-only patterns in the Discussion. 

**Table 4:** Directional classification between MMD response and patient-reported response

Response class	Patients
MMD response and at least one PROM response	209/417 (50.1%; 95% CI 45.3–54.9)
MMD response without MIDAS or HIT-6 response (frequency-only)	18/417 (4.3%; 95% CI 2.7–6.7)
MIDAS or HIT-6 response without MMD response (PROM-only)	111/417 (26.6%; 95% CI 22.6–31.1)
No MMD or PROM response	79/417 (18.9%; 95% CI 15.5–23.0)
Exact paired comparison: frequency-only vs. PROM-only	*P* < 0.001

Frequency-only response indicates MMD response without MIDAS or HIT-6 response. PROM-only response indicates MIDAS or HIT-6 response without MMD response

### Exploratory association analyses

In exploratory logistic regression, age showed an association with discordant classification and with PROM-only response. Chronic migraine status was associated with lower odds of PROM-only response in this dataset. Baseline MMD, MIDAS, and HIT-6 did not provide a simple separation between concordant and discordant patients (Table 5). These models should be read as descriptive association analyses, not as prediction models.

**Table 5:** Exploratory logistic regression models for discordance

Exploratory endpoint	Predictor	OR (95% CI)	*P* value
Directional discordance (frequency-only or PROM-only)	Age, per year	0.98 (0.96–0.99)	0.009
Directional discordance (frequency-only or PROM-only)	Female sex	0.77 (0.39–1.52)	0.450
Directional discordance (frequency-only or PROM-only)	Baseline MMD, per day	0.99 (0.94–1.04)	0.652
Directional discordance (frequency-only or PROM-only)	Baseline MIDAS, per point	1.00 (1.00–1.00)	0.491
Directional discordance (frequency-only or PROM-only)	Baseline HIT-6, per point	1.01 (0.97–1.06)	0.540
Directional discordance (frequency-only or PROM-only)	Chronic migraine	0.61 (0.34–1.09)	0.096
PROM-only response	Age, per year	0.97 (0.96–0.99)	0.003
PROM-only response	Female sex	0.78 (0.37–1.61)	0.497
PROM-only response	Baseline MMD, per day	1.01 (0.96–1.06)	0.802
PROM-only response	Baseline MIDAS, per point	1.00 (0.99-1.00)	0.479
PROM-only response	Baseline HIT-6, per point	1.03 (0.98–1.08)	0.198
PROM-only response	Chronic migraine	0.43 (0.23–0.80)	0.008

Models were descriptive and were not intended for clinical prediction

*OR* Odds ratio, *MMD* Monthly migraine days, *MIDAS* Migraine Disability Assessment, *HIT-6* Headache Impact Test-6

**Fig. 4:**
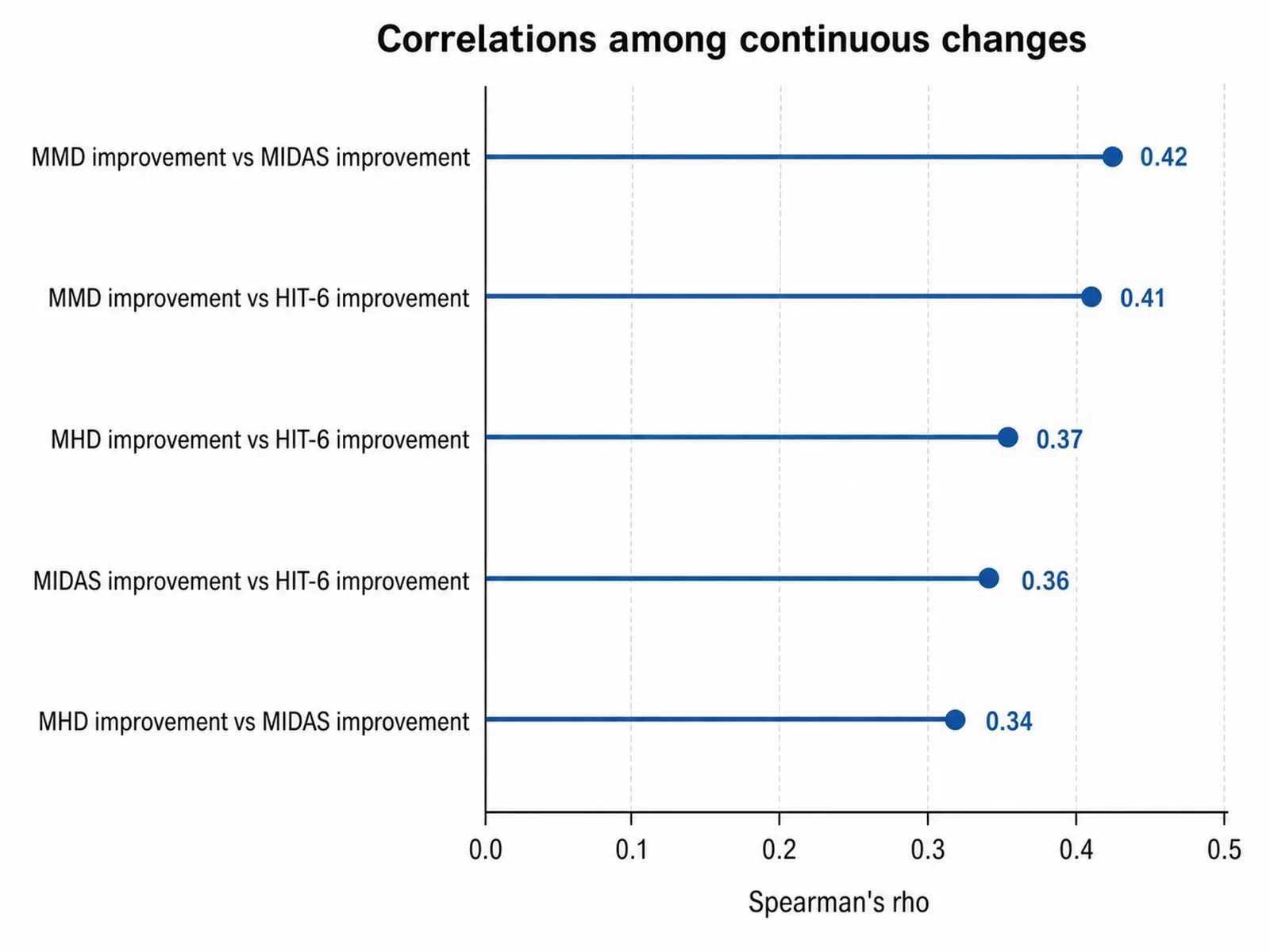
Spearman correlations among continuous improvements in MMD, MIDAS, and HIT-6. Correlations were positive but moderate, consistent with partial rather than complete overlap between domains

Continuous improvements in frequency-based and patient-reported outcomes were related, but the correlations were moderate rather than strong. MMD improvement correlated with MIDAS improvement (Spearman rho 0.42) and with HIT-6 improvement (rho 0.41). The correlation between MIDAS and HIT-6 improvement was also moderate (rho 0.36) (Table 6; Fig. 4). These continuous analyses support partial overlap between domains and caution against treating binary responder thresholds as the whole response picture. 

**Table 6:** Correlations among continuous improvements

Continuous change pair	Spearman rho	*P* value
MMD improvement vs. MIDAS improvement	0.42	< 0.001
MMD improvement vs. HIT-6 improvement	0.41	< 0.001
MHD improvement vs. MIDAS improvement	0.34	< 0.001
MHD improvement vs. HIT-6 improvement	0.37	< 0.001
MIDAS improvement vs. HIT-6 improvement	0.36	< 0.001

Positive values indicate that improvements tend to occur together. *P* values are unadjusted and should be interpreted descriptively

### Sensitivity analyses

Threshold sensitivity analyses did not remove the central pattern. Varying the MMD threshold or requiring both PROM endpoints changed the absolute number of responders, as expected, but discordance remained common and agreement remained incomplete (Supplementary Table S2). This supports the interpretation that endpoint non-interchangeability was not an artefact of one single threshold choice.

The supplementary Dryad analysis is reported only in Supplementary Table S3 and Supplementary Figure S1. It is descriptive, diary-based, and not used to address cross-domain discordance because MIDAS and HIT-6 were not available.

## Discussion

This secondary analysis asked a narrow but clinically relevant measurement question: does one responder label classify the same migraine patients across headache frequency, disability, and headache impact after CGRP-pathway monoclonal antibody treatment? In this public patient-level dataset, responder labels were not interchangeable. Response rates for MMD, MIDAS, and HIT-6 were broadly similar at the group level, but patient-level overlap was incomplete. Only about one third of patients met all three definitions, and nearly one half had at least one discordant endpoint. The results should therefore be interpreted as evidence of endpoint-dependent classification rather than as evidence that any one endpoint is superior.

The direction of discordance is clinically important. PROM-only response was much more common than frequency-only response. This finding should not be read as an argument that PROMs should replace headache diaries, but it does show that diary-based frequency thresholds and patient-reported disability or impact thresholds do not always identify the same individuals. A both-domain responder probably represents the most straightforward pattern, with improvement in both headache frequency and patient-reported burden. A frequency-only responder may have fewer migraine days but continue to experience residual disability, substantial impact on activities, persistent associated symptoms, or comorbid burden. A PROM-only responder may not cross a frequency threshold but may still experience treatment benefit that is meaningful in daily life. A neither-domain non-responder may need reassessment of treatment strategy, adherence, diagnosis, comorbidities, or treatment goals.

Several clinical explanations may account for the predominance of PROM-only response. Frequency thresholds treat all migraine days equally, whereas patients may experience improvement through shorter attacks, lower pain intensity, fewer associated symptoms, better predictability, improved response to acute medication, reduced need for rescue treatment, or less interference with work, family, social participation, and emotional functioning. Such changes can plausibly improve MIDAS or HIT-6 without producing a large enough reduction in MMD to cross the binary frequency threshold. Because the public dataset did not include detailed attack-severity, duration, acute-treatment response, missed-work context, or daily quality-of-life variables, this explanation should be regarded as clinically plausible rather than proven by the present analysis.

The continuous analyses support the same cautious interpretation. Improvements in MMD, MIDAS, and HIT-6 were correlated, so the domains are related. The correlations were only moderate, however, which is consistent with partial overlap. Response to preventive treatment occurs on a continuum, whereas responder status requires dichotomizing that continuum. A 30% or 50% threshold is useful for standardization and communication, but it is inherently imprecise at the individual level. Patients close to a cutoff may be assigned to different binary categories despite similar clinical courses, and improvement in frequency may not progress in parallel with improvement in disability or headache impact.

The non-equivalence of MMD, MIDAS, and HIT-6 thresholds is also central to interpretation. MMD is a diary-based count of migraine days. MIDAS mainly quantifies days of missed or reduced activity attributable to headache, and it may therefore track frequency-related disability more closely in some patients. HIT-6 captures a broader perception of headache impact across several domains and may change even when headache-day counts change only modestly. Part of the observed discordance is therefore likely to reflect differences in measurement construct, recall frame, scaling, and threshold definition rather than a true biological contradiction in treatment response. The combined “any PROM response” category was used only as a directional map and should not be taken to mean that MIDAS and HIT-6 are identical instruments.

Comorbidities and contextual factors may further weaken a one-to-one relationship between headache frequency and patient-reported burden. Migraine occurs within a broader neurovascular and biopsychosocial context, and autonomic symptoms, sleep disturbance, vascular risk factors such as hypertension, medication overuse, anxiety or depression, and psychosocial burden may all influence disability and perceived headache impact [26, 27]. These factors could contribute to PROM improvement or residual PROM burden independently of MMD change. The Kiel public dataset did not contain sufficiently detailed and harmonized comorbidity variables to test these mechanisms directly, and this limitation should be considered when interpreting the discordant categories.

The findings also fit with recent discussion that treatment goals in migraine should be more ambitious than partial frequency reduction alone, with attention to earlier effective treatment, patient-centered outcomes, function, and broader well-being [28–30]. The present analysis did not test early versus late treatment, treatment sequencing, or long-term well-being. Nevertheless, the discordance results are consistent with the principle that treatment evaluation should not be reduced to a single frequency threshold when patient-reported disability and impact move differently in a substantial proportion of patients.

This analysis should be distinguished from the source Kiel publication. The source study compared responder rates and examined whether single outcome measures might be sufficient for routine treatment evaluation [17]. The present analysis used the same public data for a different purpose: patient-level agreement, directional discordance, and continuous cross-domain relationships. The result is not a new treatment rule, but a clearer description of what is lost when response is reduced to a single binary endpoint.

The fMRI-related Dryad cohort was retained only in the Supplementary material. Because it lacks MIDAS and HIT-6 and was collected over a short mechanistic-study window, it cannot validate the Kiel cross-domain findings and should not be interpreted as evidence for PROM-frequency discordance. Its role is limited to documenting the provenance and descriptive early-window diary analysis of a separate public CGRP-pathway monoclonal antibody dataset [19–21].

Generalizability also requires caution. The Kiel cohort was strongly female-predominant, with only 43 male patients. This distribution is consistent with migraine epidemiology and with many specialty-care migraine cohorts, but it does not allow robust male-specific estimates of responder discordance. Sex-related differences in migraine biology, endocrine influences, symptom presentation, comorbidity patterns, treatment experience, and health-care seeking could affect the relationship between headache frequency and patient-reported disability or impact. The results are therefore most directly applicable to female-predominant, high-burden specialty-care migraine populations and should not be assumed to quantify discordance in male patients.

Other limitations should be acknowledged. The primary Kiel dataset and supplementary Dryad source were public secondary data sources, and the analyses inherit the design choices, eligibility criteria, follow-up schedules, and measurement procedures of the source studies. The Kiel data came from a tertiary headache centre and included only patients with complete outcome documentation, which may select for a population with high burden and structured follow-up. The analysis was observational and cannot establish treatment efficacy, causal pathways, mechanisms of PROM-only response, or superiority of one endpoint. Logistic regression models were exploratory, not externally validated, and should not be used for patient-level prediction. The Dryad cohort was small, had a short observation window, and lacked MIDAS and HIT-6; it should therefore remain supplementary.

Within these limits, the practical implication is to report and interpret response across complementary domains. MMD remains central because it is diary-based, clinically intuitive, and widely used in trials, guidelines, and real-world audits. However, MMD alone is an incomplete description of treatment benefit for individual patients. A patient who does not cross a frequency threshold may still experience meaningful improvement in disability or headache impact, whereas fewer migraine days may not fully resolve residual burden in another patient. Reporting both-domain, frequency-only, PROM-only, and neither-domain response can provide a more transparent account of treatment benefit without abandoning standard frequency outcomes.

## Conclusions

Responder classification after CGRP-pathway monoclonal antibody treatment varied according to whether response was defined by migraine frequency, disability, or headache impact. Frequency-based and patient-reported responses overlapped only partly, and PROM-only response was more common than frequency-only response. These findings do not establish a new responder definition or show that one endpoint is superior; they support complementary reporting of headache frequency, disability, and headache impact in future migraine studies and real-world evaluations.

## Supplementary Information

Supplementary Material 1. Supplementary Figure S1. Early-window headache-day reduction in the Dryad fMRI-related cohort.

Supplementary Material 2. Supplementary Table S1. Joint response patterns across MMD, MIDAS, and HIT-6.

Supplementary Material 3. Supplementary Table S2. Threshold sensitivity analyses.

Supplementary Material 4. Supplementary Table S3. Dryad early-window headache-diary analysis.

## Data Availability

The analysis used public anonymized datasets. The Kiel dataset is available from Zenodo [18], and the supplementary fMRI-related clinical dataset is available from Dryad [20]. Derived datasets, analysis code, tables, and figures are included in the accompanying submission package.

## Abbreviations

CGRP: Calcitonin gene-related peptide
fMRI: Functional magnetic resonance imaging
HIT-6: Headache Impact Test-6
ICHD-3: International Classification of Headache Disorders, third edition
MHD: Monthly headache days
MIDAS: Migraine Disability Assessment
MMD: Monthly migraine days
PROM: Patient-reported outcome measure
STROBE: Strengthening the Reporting of Observational Studies in Epidemiology
